# Centres of Excellence for Adolescent Health and Development: A Case Study from Uttar Pradesh, India

**DOI:** 10.3390/ijerph20043042

**Published:** 2023-02-09

**Authors:** Devika Mehra, Rahul Rajak, Sujata Deo, Qazi Najmuddin, Kshetrimayum Surmala Devi, Suresh Kumar Rathi, Sunil Mehra

**Affiliations:** 1 Department of Research and Innovation, MAMTA Health Institute for Mother and Child, New Delhi 110048, India; 2 Social Medicine and Global Health, Department of Clinical Sciences, Lund University, 20502 Malmö, Sweden; 3 Gynecology and Obstetrics Department, King George Medical University, Lucknow 226003, India; 4 Programe Division, MAMTA Health Institute for Mother and Child, Lucknow 226021, India; 5 Department of Central Research and Innovation, Sumandeep Vidyapeeth Deemed to Be University, Vadodara 391760, India

**Keywords:** adolescent, health services, counselling, referral services, centre of excellence, India

## Abstract

Adolescents and young adult comprise a significant proportion of India’s population. Although, this group of the population faces serious challenges to their health and well-being. To promote their health and well-being, Centre of Excellence (CoE) at King George’s Medical University, Lucknow, India, serves as an advanced care facility for 10–24-year-old adolescents and young adult women. This paper reports the socio-demographic characteristics of, and health services availed to adolescents and young adults who are visiting the CoE in Lucknow, India. A total of 6038 beneficiaries received clinical services during June 2018–March 2022. Out of total clinical services, 38.37% counselling and 37.53% referral services were utilised. Menstruation (46.29%), sexual and reproductive (28.19%), nutrition (5.91%), and mental health (1.67%) related problems were highly reported. The age of beneficiaries is classified into three categories, i.e., 10–14, 15–19, and 20–24 years. Prevalence of overweight was highest among adolescents aged 20–24 years compared to other age groups. Other than nutrition, late-adolescent girls (15–19) faced more health problems than their counterparts. The percentage of beneficiaries decreased significantly during and post the COVID-19 period (<0.001). Therefore, age-specific programs are currently needed, and interventions need to be designed accordingly.

## 1. Introduction

Adolescents are recognized by the World Health Organization as the age group between 10–19 years and “young” as those in the 10–24 year age group [1]. The estimates suggest that one-fifth of the world’s adolescents and young people reside in India [2]. It is a period of physical, hormonal, physiological, psychological, and behavioral changes. Adolescence is the transition from dependency to autonomy [3]. During this phase, adolescents and young people are exposed to various physical and mental health transitions [4]. According to Global health estimates (2016), in India, the adolescent mortality rate accounted for 844 deaths per 1,000,000 people [5]. Additionally, in India, adolescents and young women face many social cultural issues such as child marriage and early child bearing, which may result in many sexual and reproductive health (SRH) and nutritional issues. Therefore, promoting a healthy and disease free lifestyle during this phase and investing in adolescent and young people is key to reaching a better demographic dividend for the country [4]. Adolescents are part of many Sustainable Development Goals (SDGs) which are associated with reproductive health, nutrition, sexual and intimate partner violence (IPV), child marriage, education, and employment [6]. However, the health status of adolescent and young adults is still poor.

National Family Health Survey (NFHS)-5 (2019–2020) highlights that the prevalence of pregnant women in the country is 6.8% [7]. Also, 32% of the girls are from early adolescence (10–14 years), and 48% of the girls from late adolescence and (15–19 years) are anemic [8]. National representative data also shows that 1.5% of young women aged 18–29 years have experienced sexual violence [7]. As per the National Mental Health Survey (2015–2016), 7% prevalence of psychiatric disorders among 13–17 year olds occurs among both the genders [9]. Depression among adolescents was high in the late adolescent age group (15–19) at 11.7%, as compared with that in early adolescence (10–14), i.e., 8.9% [10]. These statistics show the vulnerability among adolescents that constitute barriers for their growth and development [11].

Additionally, access to sexual and reproductive health (SRH) information and services has always been socially stigmatized and scrutinized for adolescent and young adult women [12]. They face various menstrual problems such as dysmenorrhea, premenstrual syndrome, menorrhagia, and irregular cycle [13]. Poor dietary behaviors may contribute to nutrition-related problems that have consequences for long-term health problems.

In India, comprehensive health services for adolescent and young adult women are still inadequate and unfocused. There is limited access to information on issues such as SRH, mental health, non-communicable diseases (NCD), violence against adolescents and young adult women; therefore, this portion of the population remains a neglected group. Additionally, India traditionally has large groups which are from the most disadvantaged socio-economic backgrounds, called scheduled castes (SCs) and scheduled tribes (STs). There is a considerably large gap between health accessibility, availability, and affordability among adolescent girls of SCs/STs and the rest of the population [14,15]. Therefore, there is a need to reduce this gap by providing health facilities specifically for adolescent and young adult women. The majority of adolescent and young adult-oriented schemes address only reproductive and sexual health-related issues.

To ensure the holistic development of adolescents and young adults, the Ministry of Health and Family Welfare, Government of India, launched the Rashtriya Kishor Swasthya Karyakram (RKSK), also known as the National Adolescent Health Programme (NAHP), in 2014. Initially, RKSK program focused on SRH only, but later included NCD, nutrition, mental health, substance abuse, injuries, and violence [16]. In a review by the WHO, the RKSK program was reported to be successful in four states, namely Haryana, Madhya Pradesh, Maharashtra, and Uttarakhand, in terms of establishing adolescent-friendly health clinics up to primary health centre level, resource allocation for community-level activities, and continuous improvement in community engagement at state, district and block level [16].

To address the adolescent and young adult health issues, the Government of Uttar Pradesh recognized the need, and associated as a partner with MAMTA Health Institute for Mother and Child (MAMTA HIMC) to establish pioneering Centres of Excellence for Adolescent Health and Development (CoE-AHD) at King George’s Medical University (KGMU), Lucknow and Sir Sunderlal Hospital under the Banaras Hindu University, Varanasi, India. These centres are one of the first and unique centres that provide comprehensive care for adolescents and young adults. The CoE-AHD has focused on RKSK thematic areas and highlighted adolescent health epidemiology in six major domains (Nutrition, SRH, Mental Health, Injuries and Violence, Substance Abuse, Screening of Non-communicable Diseases–NCDs). The CoE-AHD not only serves as an advanced healthcare facility, but also conducts operational and intervention research which provides evidence and promotes advocacy for policy changes related to adolescent health.

This paper focuses on the services availed to adolescent and young adult (10–24 years) females who have visited the CoE-AHD in Lucknow, India. The overall aim of this analysis was to investigate which health services are being utilized (counselling, clinical and referral) by adolescents and young adults, along with their socio-demographic characteristics. We also aim to generate evidence to work towards strengthening the ongoing adolescent programs such as RKSK, initiated by the Government of India, as well as increase utilization of the CoE-AHD services among adolescents and young adults.

## 2. Materials and Methods

### 2.1. Design and Setting

The current retrospective analysis is based on medical records of all beneficiaries of the Centre of Excellence (CoE-AHD) at KGMU, Lucknow, India from 1 June 2018, to 31 March 2022. This Centre is setup by the Government of Uttar Pradesh, India, in collaboration with MAMTA-Health Institute for Mother and Child (MAMTA HIMC) to support evidence-based programs for adolescent health and development in the State. As this centre has been set up in the Gynecology Department of KGMU, Lucknow, the beneficiaries visiting the CoE-AHD are mostly female. Additionally, it is important to mention that this analysis was not a medical experiment and did not require approval from the bioethics committee. Before counselling and clinical examination, all the beneficiaries agreed to share their details. Health practitioners took consent before the health examination.

The female population of Lucknow as per the 2011 census was 2.1 million. The current analysis includes girls and young females only. We have also included the beneficiaries between the age group of 10–24 years. The number of boys and male beneficiaries was negligible in the centre, hence they were excluded them from the analysis.

### 2.2. Sample Size and Distribution

A total of (*n* = 6038) beneficiaries in the age group of 10–24 years visited from June 2018–March 2022. The sample was classified into three age groups, i.e., 10–14, 15–19, and 20–24 years. Each beneficiary has been provided with a health card. To understand the status of beneficiaries between pre- and post-COVID-19 period, data were further categorized into two broad categories:

- Pre-COVID-19 phase: 1 June 2018–30 April 2020
- During and Post COVID-19 phase: 1 May 2020 to 30 March 2022

### 2.3. Socio-Demographic Profile

The socio-demographic characteristics of beneficiaries were identified by nine questions related to the following aspects: age, educational background, marital status, number of children, religion, caste, Below Poverty Line (BPL) card holder, occupation, and substance abuse.

### 2.4. Anthropometric and Physiological Parameters 

All the beneficiaries underwent a comprehensive clinical examination. At first, body weight and height were recorded. The body mass index (BMI) was computed as follows: BMI = weight (kg)/height (m^2^). The beneficiaries were categorized based on their BMI using the WHO International Standard [15]. The physiological measurements taken were systolic blood pressure (SBP) and diastolic blood pressure (DBP).

### 2.5. Health Assessment

#### 2.5.1. Counselling Services

In the CoE-AHD, all the beneficiaries availed themselves of the counseling service with a particular focus on RKSK domains:

- Sexual and Reproductive Health
- Menstruation
- Nutrition
- Non-Communicable Diseases (NCD) screening
- Mental health
- Violence (including injuries)

#### 2.5.2. Clinical Examination and Referral Services

**Figure 1:**
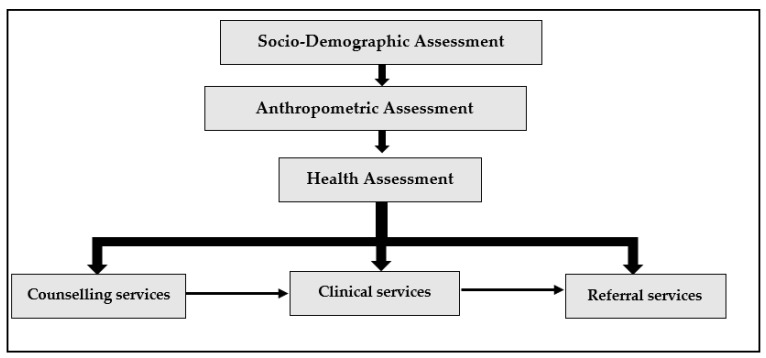
Flowchart for the Beneficiaries in the centre used for assessment by health professionals and doctors.

CoE-AHD offers beneficiaries comprehensive diagnostic clinical services by a team of qualified health professionals. Further, if doctors felt the need for further check-up and diagnosis, then beneficiaries were further referred to specialized doctors as shown in Figure 1 below.

### 2.6. Data Processing

Statistical Package for Social Sciences (SPSS) version 23.0 software was used for data processing and analysis. Descriptive statistical methods were used to summarize the socio-demographic information of the beneficiaries. The continuous data were expressed as “mean ± standard deviation (SD)” and discrete data as “frequency and percentage”. To examine the significant changes in the beneficiaries and health issues between pre-COVID-19 and during and post-COVID-19 period, chi-squared test was used. The association was considered significant at *p*-value < 0.05.

## 3. Results

### 3.1. Socio-Economic and Demographic Profile of the Beneficiaries

Table 1 summarizes the socio-demographic characteristics of the analyzed population. A total of 6038 adolescents and young adult females visited the CoE-AHD between 2018 and 2022. The majority of the beneficiaries that visited the centre were in the age group of 15–19 years (55.0%), followed by 20–24 years (38.2%), and 10–14 years (6.8%) age group. The mean age of the beneficiaries was 18.94 ± 2.72 years. The proportion of 15–19-year-olds was high compared to beneficiaries in the other age groups in the analysis period. There was an increasing trend in the number of visitors from 2018 (2.9%) to 2022 (12.3%) among Schedule caste and Schedule tribe females. Education-wise analysis reveals that out of total beneficiaries, 36% were graduates, and 14.7% had never enrolled in school. More than three fourth of the participants (77.0%) were unmarried. The majority of the respondents were from non-working categories (97.9%).

### 3.2. Physiological and Anthropometric Characteristics of the Beneficiaries

Physiological and anthropometric characteristics such as height, weight, blood pressure, and BMI from 2018–2022 (up to march) are shown in Table 2. Analysis shows that mean height and weight were high among young adults (20–24) across the observation years. The overall mean height among 20–24 years was (152.58 ± 3.34) and weight (49.79 ± 6.99), respectively. Regarding BMI, early adolescent (10–14) girls had low BMI (16.94 ± 3.41) compared with late adolescent and young adult females. Overall, an increase in the mean of physiological and anthropometric parameters was seen in the late age of adolescent and young adult females (Table 2).

### 3.3. Distribution of Body Mass Index by Different Age Groups

Table 3 illustrates the distribution of beneficiaries by BMI during 2018–2022. The prevalence of underweight was highest among 15–19-year-olds across all years. Across age groups, the highest prevalence of obesity was observed among adolescents aged 20–24, i.e., 2018 (78.50%, *p*-value: <0.001), 2019 (46.50%, *p*-value: <0.01), 2020 (53.50%, *p*-value: <0.01), 2021 (54.20%, *p*-value: <0.01), 2022 (78.1%, *p*-value: <0.001).

### 3.4. Distribution of Counselling, Clinical, and Referral by Year and Age Group

Table 4 shows the distribution of counselling, clinical, and referral by year and age group. We observed that late adolescent girls (15–19 years) received the highest counselling services (*n* = 3319) compared with other age groups. On the other hand, only 410 beneficiaries from early adolescent age group (10–14) received counselling services. We found that out of total counselling, 41.06% of the beneficiaries further availed themselves of clinical services. It was also observed that those beneficiaries who received clinical services were further referred to appropriate departments.

**Figure 2:**
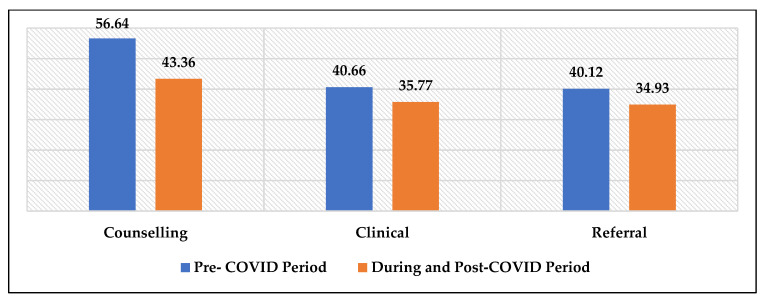
Distribution of counselling, clinical, and referral of beneficiaries for Pre- and during and Post-COVID-19 Period. Note: Pre-COVID-19 phase: 1 June 2018–30 April 2020; During and Post-COVID-19 phase: 1 May 2020 to 30 March 2022.

Figure 2 shows changes in the beneficiaries’ services during pre- and post-COVID-19 periods. We found that all three services (counselling, clinical, and referral) decreased from the pre-COVID-19 period to the post-COVID-19 period. For instance, the counselling services were 56.64% during the pre-COVID-19 period and 43.09% during and after the COVID-19 period.

### 3.5. Year-Wise Prevalence of Beneficiary Health Problems

**Figure 3:**
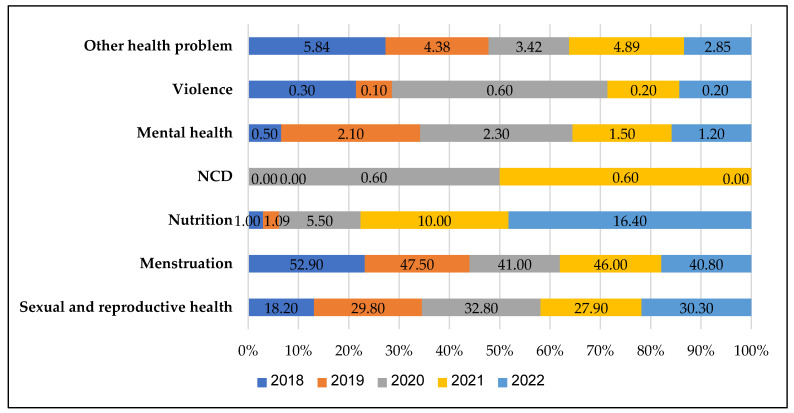
Year-wise counselling of beneficiaries by their health problems. Note: NCD: Non communicable diseases. NCD not reported in the 2018, 2019, and 2022 year.

In Figure 3, we found that beneficiaries mainly visit the facilities for sexual and reproductive health, menstruation, nutrition, NCD, mental health, violence, and other health problems. Counselling related to menstruation was most commonly reported across the year. Sexual and reproductive health issues were the second most common reported counselling, followed by nutrition-related issues. Mental health and violence-related counselling were rarely reported. Other health problems (body ache, cough, fever, etc.) were also observed during the analysis period.

### 3.6. Prevalence of Health Problems among Early Adolescents, Late Adolescents, and Young Beneficiaries

We found that amenorrhea, dysmenorrhea, and white discharge were the most frequent health problems reported by all beneficiaries. Within the sexual and reproductive health (SRH) problems reported by beneficiaries, white discharge (12.89%), ovarian cyst and its related problems (5.63%), bleeding, and pain (3.54%) were frequently reported. For menstruation-related problems, a total of 2795 cases were reported. Out of that, dysmenorrhea and amenorrhea (17.27% each) were the most common menstruation reported problems and highly observed in early and late adolescent girls (10–19 Years). Only 12 cancer patients visited the CoE for cancer-related symptoms. Under the mental health issues, depression, behavioral disorders, learning problems, and emotional disorders were reported, but the cases were less observed. In the nutrition issues, Ante Natal Care (5.13%), anemia (0.46%), and iodine deficiency (0.31%) were reported. Other health problems, such as body ache (15.42%) etc. were also reported (Table 5).

Beneficiaries visited the CoE-AHD for all health-related issues during the pre- and post-COVID-19 period reported in Table 6. A total of 56.64% beneficiaries visited for counselling during the pre-COVID-19 period and 43.36% during and post-COVID-19 period. We found a significant decrease in beneficiaries who visited the CoE-AHD for treatment (<0.001). For example, sexual and reproductive health problems (<0.001), menstruation-related problems (<0.001), nutrition (<0.05), and other health problem (<0.001) have significantly decreased during and in the post-COVID-19 period.

## 4. Discussion

The Centre of Excellence for Adolescent Health and Development is the Government of Uttar Pradesh and MAMTA’s flagship intervention that provides adolescent-friendly health services. The present analysis focuses on adolescent and young adult females who visited the CoE-KGMU from June 2018–March 2022. We summarized our findings in three services (counselling, clinical, referral) in line with the objective of the analysis. The learnings from this CoE would strengthen the ongoing RKSK program initiated in the State, spearheaded by the National program implementation of the Government of India. The strength of the RKSK program has been its health promotion approach; there has been a paradigm shift from the existing curative clinic-based services to promotion and prevention by reaching adolescents in their own environment, such as schools and communities. The school-based interventions have increased outreach of preventive health and its messages, and influenced utilization of services in these centres (CoE). Additionally, it has also increased referral services from adjoining districts. Eight papers have already been published to strengthen state specific programming. The initiative has also led to an inter-sectoral approach through the state health program where different departments have now come together to improve adolescent health.

However, holistically, adolescent health is still at a nascent stage in the country. Even though RKSK was launched in 2014, it started rolling out in the states only in 2016. It has been only five years since its implementation started, and the last two years of service were affected by the COVID-19 pandemic. In this analysis we have shown how the outbreak of the COVID-19 pandemic, and the subsequent lockdown in 2021, decreased the number of beneficiaries from the pre-COVID-19 period to the post-COVID-19 period. During the counselling services, we observed that very limited numbers of SC/ST caste-based beneficiaries visited the CoE-AHD. A similar finding was observed in a study conducted in three states of India (Bihar, Chhattisgarh, and Odisha) among pregnant adolescent girls, which found that SC/ST adolescents were less likely to use health care services than other castes [17].

The analysis shows that SRH and menstruation-related problems were most prevalent during 2018–2022. The highest prevalence of SRH and menstruation-related problems were reported in the late adolescent age group (15–19 years), as compared to early adolescent and young adult females. This finding was supported by another study that focused on adolescents and young adults, which found that 78.3% of adolescent females already had coital initiation, which could be a major reason for the SRH issues among late adolescent girls (15–19 years) [18]. Prior studies have reported that adolescents who were married at a very young age were more likely to have SRH complications than those married as adults [19].

The current analysis also found that among SRH problems, white discharge (12.89%), ovarian cyst (5.63%), and bleeding and pain (3.54%) were major issues that were reported. A similar observation was found in a hospital-based retrospective study among adolescents (10–19 years), that white discharge was the most common reproductive health problem among adolescent girls [20]. A significant reduction in counselling for SRH problems (<0.001), menstruation-related problems (<0.001), nutrition (<0.005), and other health problems (<0.001) from the pre-COVID-19 period to the post-COVID-19 period was observed. The cases of mental health problems were also observed in the pre- and during and post-COVID-19 period. Our finding was in line with the previous study findings that during the COVID-19 pandemic, nearly 27.5% of adolescent girls from central India between 15 and 20 years had high levels of psychological distress [21]. A previous study also reported that during the COVID-19 period, young girls had chronic shortages of sanitary napkins, and their difficulties in accessing the napkins increased significantly. These kinds of crises were one of the primary causes of SRH problems among adolescents [12].

Another major finding of this analysis is that ANC check-ups were majorly reported, followed by anemia and iodine deficiency. A previous study reports that those adolescent girls consuming IFA tablets were less likely to be anemic compared to those not consuming IFA tablets (OR = 0.09, *p* < 0.001) [22]. Under mental health problems, the prevalence of depression was observed more in late adolescent and young adult women. There is prior evidence which shows that depression and stress are more prevalent among school-going adolescent girls [23]. Therefore, there is a need for scalable mental health strategies, especially for the late adolescent age group and young adults.

A total of 12 cancer cases were reported during the analysis period. All cancer cases were reported in the late adolescent and young women age group. In contrast to our findings, a previous study found that prevalence of cancer was higher among the 24–30 age group compared to adolescents and young adults (10–24) [24]. It may be because our analysis focused only on adolescent and young women. Furthermore, vomiting, chest pain, weakness, and liver-related problems were frequently reported health issues by adolescent and young beneficiaries. Our analysis also found that a negligible number of beneficiaries were currently using any kind of substance. This finding is in congruence with a previous study conducted by Global Adult Tobacco Survey in Uttar Pradesh, which found that less than 3.2% of women currently smoke tobacco [25]. In our data we found that a very small proportion of adolescent girls and young adult women came to the hospital for violence-related issues, which could be due to the stigma related to reporting violence and its after effects.

### Strengths and Limitations

A major strength of this data is the robust sample size which gives us an estimation of the general adolescent health problems. The availability of strong data for adolescent and young adult women would help policy-makers and researchers to strengthen the adolescent health system. Another strength of the analysis was the retrospective data from the last 5 years which covered all the RKSK themes, especially in pre-COVID, during and post-COVID times. A limitation was that we could not measure all possible health aspects of adolescent and young adult health. Another limitation was that the current analysis focuses only on females; therefore, problems related to boys were not reflected. Lastly, this analysis was based on only one health facility, KGMU, Lucknow, India; therefore, the result does not reflect the actual scenario of the state or region.

## 5. Conclusions 

Adolescents and young adult women face many SRH problems. Therefore, there is a need to have access to timely, integrated, high-quality, multi-disciplinary SRH health services to ensure effective assessment, treatment, and support. In terms of age-wise health effects, late adolescent girls were more vulnerable. Therefore, it is essential to develop age-specific screening tools. To achieve comprehensive health care for this population, we need to have a multi-dimensional approach covering various aspects of health problems, with a particular emphasis on SRH and mental health. Additionally, the scientific rigor of the tools needs be have more precision and needs to be designed for specific diseases to get real estimates.

Adolescent and young adults (10–24 years of age) are highly recommended for promoting the RKSK services throughout India. The findings also urge the government to invest in adolescent and young adult health programs across India. There is a need to include SRH and menstruation-related education in the school teaching curriculum that will help in the prevention and promotion of reproductive and sexual health-related problems. At the same time, it will also help in combatting the stigma related to it. There is also a need to educate parents regarding the nutritional requirements, and to adopt a diet appropriate for adolescents and young adults.

We should aspire to scale up such adolescent-friendly health centres in different states of India. This will further help in early detection of adolescent health problems. We need to enable the visit of SC/ST beneficiaries in the CoE-AHD adolescent-specific health awareness program, which must be included in the hospital-based curriculum. Counselling and screening of adolescent and young adult females should be done on a regular basis so that it can be an effective strategy to control the existing diseases and to update the occurrence of any new disease. Additionally, there is a need to include adolescent and young adult males in CoE-AHD, KGMU. It will help to address the adolescent-related problems more comprehensively. Gender inclusive interventions are important to improve health outcomes.

## Figures and Tables

**Table 1:** Socio-demographic characteristics of the beneficiaries (*n* = 6038).

Background Characteristics	2018	2019	2020	2021	2022	Total N (%)
Age category						
10–14 Years	56 (7.1)	183 (9.1)	69 (7.8)	80 (4.1)	22 (5.2)	410 (6.8)
15–19 Years	297 (37.6)	1282 (64.0)	520 (59.1)	1065 (54.8)	155 (36.7)	3319 (55.0)
20–24 Years	437 (55.3)	536 (26.9)	291 (33.1)	797 (41.0)	245 (58.1)	2309 (38.2)
Mean (S.D)	19.6 (3.15)	18.1 (2.63)	18.7 (2.63)	19.28 (2.41)	20 (2.85)	18.94 (2.72)
Marital status						
Married	279 (35.3)	293 (14.6)	225 (25.6)	464 (23.9)	128 (30.3)	1389 (23.0)
Unmarried	511 (64.7)	1711 (85.4)	655 (74.4)	1478 (76.1)	294 (69.7)	4649 (77.0)
Having children						
No	257 (92.1)	261 (89.1)	206 (91.6)	463 (99.8)	128 (100.0)	1315 (94.7)
Yes	22 (7.9)	32 (10.9)	19 (8.4)	1 (0.2)	0 (0.0)	74 (5.3)
Religion						
Hindu	675 (85.4)	1631 (81.4)	721 (81.9)	1665 (85.7)	334 (79.1)	5026 (83.2)
Other *	115 (14.6)	373 (18.6)	159 (18.1)	277 (14.3)	88 (20.9)	1012 (16.8)
Caste						
General	753 (95.3)	1870 (93.3)	646 (73.4)	1348 (69.4)	322 (76.3)	4939 (81.8)
SC/ST	23 (2.9)	111 (5.5)	109 (12.4)	194 (10.0)	52 (12.3)	489 (8.1)
Other	14 (1.8)	23 (1.1)	125 (14.2)	400 (20.6)	48 (11.4)	610 (10.1)
BPL Card holder						
Yes	7 (0.9)	55 (2.7)	50 (5.7)	29 (1.5)	4 (0.9)	145 (2.4)
No	783 (99.1)	1949 (97.3)	830 (94.3)	1913 (98.5)	418 (99.1)	5893 (97.6)
Education						
No Schooling	63 (8.0)	249 (12.9)	147 (16.7)	359 (18.5)	70 (16.6)	888 (14.7)
Primary	158 (20.0)	517 (25.8)	209 (23.8)	293 (15.1)	63 (14.9)	1240 (20.50)
Secondary	203 (25.7)	754 (37.6)	230 (26.1)	435 (22.4)	114 (27.0)	1736 (28.8)
Graduate	366 (46.3)	484 (24.2)	294 (33.4)	855 (44.0)	175 (41.5)	2174 (36.0)
Occupation						
Employed	6 (0.8)	13 (0.6)	15 (1.7)	73 (3.8)	20 (4.7)	127 (2.1)
Unemployed	784 (99.2)	1991 (99.4)	865 (98.3)	1869 (96.2)	402 (95.3)	5911 (97.9)
Substance use						
No	790 (100)	2003 (99.9)	879 (99.9)	1939 (99.8)	422 (100)	6033 (99.9)
Yes	0 (0.0)	1 (0.1)	1 (0.1)	3 (0.2)	0 (0.0)	5 (0.1)

Note: BPL—Below Poverty Line; SC—Schedule caste; ST—Schedule Tribe; * Other: Islam, Jainism.

**Table 2:** Physiological and anthropometric characteristics of the beneficiaries (*n* = 6038).

Variables	Age Group (Years)	2018	2019	2020	2021	2022	Total
		Mean ± SD	Mean ± SD	Mean ± SD	Mean ± SD	Mean ± SD	Mean ± SD
Height	10–14	148.19 (5.70)	143.06 (8.55)	144.85 (9.20)	143.66 (8.32)	143.68 (8.35)	144.21 (8.41)
	15–19	151.44 (2.69)	150.93 (3.50)	152.03 (3.86)	151.75 (4.14)	151.95 (4.40)	151.26 (3.77)
	20–24	152.24 (2.08)	151.89 (3.00)	153.21 (3.37)	152.87 (3.69)	153.02 (4.27)	152.58 (3.34)
Weight	10–14	37.54 (10.35)	34.41 (8.82)	35.4 (8.03)	36.03 (9.50)	39.59 (10.26)	35.60 (9.19)
	15–19	45.5 (6.81)	44.6 (6.19)	45.81 (6.19)	48.05 (6.37)	47.61 (6.99)	46.07 (6.82)
	20–24	48.47 (8.31)	49.32 (6.10)	49.32 (6.08)	50.76 (6.97)	50.83 (6.77)	49.79 (6.99)
SBP * (mm of Hg)	10–14	99.76 (8.85)	102.40 (10.07)	103.63 (7.57)	101.23 (7.00)	103.3 (7.95)	102.12 (8.92)
	15–19	103.4 (9.43)	111.03 (10.70)	108.15 (8.11)	109.63 (8.28)	108.17 (6.40)	109.45 (9.49)
	20–24	103.86 (11.04)	111.74 (10.08)	111.63 (8.69)	110.89 (7.29)	111.78 (7.65)	110.25 (9.27)
DBP ** (mm of Hg)	10–14	77.74 (12.27)	76.24 (11.37)	73.54 (10.22)	67.41 (8.15)	66.65 (7.84)	73.75 (11.23)
	15–19	82.23 (12.16)	83.07 (10.45)	78.95 (8.80)	74.42 (8.91)	69.47 (7.33)	79.13 (18.76)
	20–24	80.73 (12.41)	85.14 (9.92)	81.91 (9.00)	74.73 (8.25)	72.11 (7.33)	78.84 (10.59)
BMI ***	10–14	16.95 (4.22)	16.65 (3.15)	16.71 (2.46)	17.43 (3.887)	18.91 (3.95)	16.94 (3.41)
	15–19	19.79 (2.81)	19.47 (2.78)	19.83 (2.50)	20.86 (2.55)	20.57 (2.64)	20.06 (2.72)
	20–24	21.26 (3.47)	21.01 (2.62)	20.96 (2.43)	21.72 (2.95)	21.73 (2.70)	20.35 (2.90)

Note: * SBP—Systolic Blood Pressure, ** DBP—Diastolic Blood Pressue, *** BMI—Body Mass Index.

**Table 3:** Year-wise distribution of body mass index by different age group.

Years	BMI Category	10 to 14	15 to 19	20 to 24	Total χ^2^
2018	Underweight	45 (23.40)	83 (43.20)	64 (33.30)	192 (24.3)
	Normal weight	8 (1.80)	183 (41.60)	249 (56.60)	440 (55.7) 1.449 ***
	Overweight	3 (1.90)	31 (18.60)	124 (78.50)	158 (20.0)
2019	Underweight	139 (21.60)	433 (67.30)	71 (11.00)	643 (32.1)
	Normal weight	38 (3.30)	739 (64.60)	367 (32.10)	1144 (57.1)
	Overweight	6 (2.80)	110 (50.70)	101 (46.50)	217 (10.8) 2.716 **
2020	Underweight	53 (22.60)	145 (61.70)	37 (15.70)	235 (26.7)
	Normal weight	16 (2.90)	328 (60.30)	200 (36.80)	544 (61.8)
	Overweight	NR *	47 (46.50)	54 (53.50)	101 (11.05) 1.288 **
2021	Underweight	56 (20.2)	156 (56.3)	65 (23.5)	277 (14.3)
	Normal weight	21 (1.60)	745 (57.3)	534 (41.1)	1300 (66.9)
	Overweight	3 (0.80)	164 (44.90)	198 (54.20)	365 (18.8) 2.481 **
2022	Underweight	12 (21.4)	27 (48.2)	17 (30.4)	56 (13.3)
	Normal weight	8 (2.7)	114 (38.9)	171 (58.4)	293 (69.4) 53.526 ***
	Overweight	2 (2.7)	14 (19.2)	57 (78.1)	73 (17.3)

Note: *** *p* < 0.001, ** *p* < 0.01, * NR: Not reported.

**Table 4:** Distribution of counselling, clinical, and referral of beneficiaries by year and age group.

**10–14 Years Age Group**			
**Year**	**Total Number of Counselling**	CS * out of Total Counselling	RS * out of Total Counselling
2018	56	29 (51.79)	27 (48.21)
2019	183	57 (31.14)	57 (31.14)
2020	69	26 (37.68)	26 (37.68)
2021	80	34 (42.50)	33 (41.25)
2022	22	10 (45.45)	8 (36.36)
Total	410	156 (38.05)	151 (36.83)
**15–19 Years Age Group**			
**Year**	**Total Number of Counselling**	CS * out of Total Counselling	RS * out of Total Counselling
2018	297	163 (54.88)	163 (54.88)
2019	1282	397 (30.96)	397 (30.96)
2020	520	196 (37.69)	196 (37.69)
2021	1065	393 (36.90)	393 (36.90)
2022	155	64 (41.29)	46 (29.67)
Total	3319	1213 (36.55)	1195 (36.00)
**20–24 Years Age Group**			
**Year**	**Total Number of Counselling**	CS * out of Total Counselling	RS * out of Total Counselling
2018	437	293 (67.04)	291 (66.59)
2019	539	176 (32.65)	176 (32.65)
2020	291	99 (34.02)	99 (34.02)
2021	797	284 (35.63)	284 (35.63)
2022	245	96 (39.18)	70 (28.57)
Total	2309	948 (41.06)	920 (39.84)

Note: CS *: Clinical services; RS *: Referral services.

**Table 5:** Distribution of beneficiaries by their health problems (*n* = 6038).

Health Problems	10 to 14		15 to 19		20 to 24		Total	
	N	%	N	%	N	%	N	%
Sexual and Reproductive health problems								
White discharge	55	13.41	374	11.27	349	15.11	778	12.89
Ovarian cyst and its related problem	21	5.12	186	5.60	133	5.76	340	5.63
Bleeding and Pain	15	3.66	136	4.10	63	2.73	214	3.54
Family planning	0	0.00	75	2.26	94	4.07	169	2.80
Vaginal Infection	18	4.39	33	0.99	44	1.91	95	1.57
Breast Related Problem	15	3.66	42	1.27	13	0.56	70	1.16
Abortion	NR *	-	18	0.54	18	0.78	36	0.60
Menstruation related problem								
Dysmenorrhea	99	24.15	712	21.45	232	10.05	1043	17.27
Amenorrhea	114	27.80	651	19.61	278	12.04	1043	17.27
Oligomenorrhea	7	1.71	212	6.39	194	8.40	413	6.84
PCOD	0	0.00	81	2.44	67	2.90	148	2.45
Hypermenorrhoea	2	0.49	78	2.35	68	2.94	148	2.45
Non-communicable diseases								
Cancer	NR *	-	5	0.15	7	0.30	12	0.20
Nutrition								
ANC	NR *	-	89	2.68	221	9.57	310	5.13
Anaemia	10	2.44	15	0.45	3	0.13	28	0.46
Iodine Deficiency-	7	1.71	4	0.12	8	0.35	19	0.31
Mental health problem								
Depression	2	0.49	28	0.84	13	0.56	43	0.71
Behavioural disorder	3	0.73	10	0.30	18	0.78	31	0.51
Learning problem	11	2.68	7	0.21	4	0.17	22	0.36
Emotional disorder	1	0.24	2	0.06	2	0.09	5	0.08
Violence		0.00				0.00		0.00
Physical violence	2	0.49	5	0.15	5	0.22	12	0.20
Sexual abuse	NR *	-	2	0.06	1	0.04	3	0.05
Other health problem								
Bodyache	NR *	-	469	14.13	462	20.01	931	15.42
** Other problem	28	6.83	85	2.56	12	0.52	125	2.07
Total	410	100.0	3319	100.0	2309	100.0	6038	100.0

Note: * NR: Not Reported, ** Other problem: Vomiting and fever, chest pain, weakness, liver related problem

**Table 6:** Comparison of prevalence of beneficiary functionality during and after the COVID-19 epidemic (*n* = 6038).

Health Issues	Pre COVID-19 Period		During and Post COVID-19 Period		*p* Value
	N	%	N	%	
Sexual and reproductive health problem	899	52.82	803	47.18	<0.001
Menstruation related problem	1704	60.97	1091	39.03	<0.001
Non-communicable diseases	7	58.33	5	41.67	0.9
Nutrition	189	52.94	168	47.06	<0.05
Mental health problem	53	52.48	48	47.52	0.9
Violence	8	53.33	7	46.67	0.6
Other health problem	560	53.03	496	46.97	<0.001
Total	3420	56.64	2618	43.36	<0.001

## Data Availability

The dataset for the adolescents and young adult women generated and analyzed during the current analysis is available from the authors upon reasonable request.
